# Analytical performance and method comparison of a quantitative point-of-care immunoassay for measurement of bile acids in cats and dogs

**DOI:** 10.1177/1040638720968784

**Published:** 2020-10-28

**Authors:** Kristina Weiler, Katharina Kleber, Sabine Zielinsky, Andreas Moritz, Natali Bauer

**Affiliations:** Department of Veterinary Clinical Sciences, Clinical Pathology and Clinical Pathophysiology, Justus-Liebig-University, Giessen, Germany

**Keywords:** bile acids, cats, dogs, point-of-care analyzer, precision, total allowable error

## Abstract

Point-of-care analyzers (POCAs) for quantitative assessment of bile acids (BAs) are scarce in veterinary medicine. We evaluated the Fuji Dri-Chem Immuno AU10V analyzer and v-BA test kit (Fujifilm) for detection of feline and canine total serum BA concentration. Results were compared with a 5th-generation assay as reference method and a 3rd-generation assay, both run on a bench-top analyzer. Analytical performance was assessed at 3 different concentration ranges, and with interferences. For method comparison, samples of 60 healthy and diseased cats and 64 dogs were included. Linearity was demonstrated for a BA concentration up to 130 µmol/L in cats (*r* = 0.99) and 110 µmol/L in dogs (*r* = 0.99). The analyzer showed high precision near the lower limit of quantification of 2 µmol/L reported by the manufacturer. Intra- and inter-assay coefficients of variation were < 5% for both species and all concentrations. Interferences were observed for bilirubin (800 mg/L) and lipid (4 g/L). There was excellent correlation with the reference method for feline (*r_s_* = 0.98) and canine samples (*r_s_* = 0.97), with proportional biases of 6.7% and −1.3%, respectively. However, a large bias (44.1%) was noted when the POCA was compared to the 3rd-generation assay. Total observed error was less than total allowable error at the 3 concentrations. The POCA reliably detected feline and canine BA in clinically relevant concentrations.

## Introduction

In dogs and cats, an increase in pre- or post-prandial serum bile acid (BA) concentration is a valuable indicator of liver dysfunction caused by various hepatic, biliary, or portal disorders that limit hepatic portal blood flow, hepatocellular uptake, or decrease BA excretion in bile.^6,7,23,24^ Several studies underline the diagnostic value of BA in dogs and cats with portosystemic shunts,^1,6,17,28,31^ with reported sensitivities of 89–100% and specificities of 67–71%.^17,31^

To date, several generations of assays are available for assessment of serum BA concentrations. The enzymatic method, also termed a 3rd-generation assay (3GA), requires manual reconstitution of lyophilized material and is therefore used mainly in small laboratories. The reaction is based on oxidation of BA that further catalyzes enzyme reactions resulting in formation of a formazan dye, which can be detected at a wavelength of 540 nm.^12^

In contrast, the enzyme cycling method (5th-generation assay, 5GA) is performed with liquid reagents, does not require manual steps, and therefore is used widely in large clinical laboratories. In the 5GA BA assay, signal amplification is achieved by repeated oxidation and reduction of BA by the enzyme 3-ɑ-hydroxysteroid dehydrogenase resulting in accumulation of reduced coenzyme thio-NADH, which is measured at a wavelength of 405 nm.^39^ Given the amplification steps, the test requires less sample volume than conventional 3GAs for BA, has higher detection sensitivity, and is considered to have superior analytical performance.^12^

A promising technology with high sensitivity for detection of small quantities of an analyte has been developed using surface plasmon resonance (SPR) and surface plasmon enhanced fluorescence (SPF).^26,38^ Laser irradiation is performed at an optimal angle onto a thin metallic film to generate a resonance reaction by induction of evanescent waves on the film that resonate with compressional waves of free electrons, named surface plasmons, to generate surface plasmon enhanced resonance. With the near-field light generated in SPR, fluorescence particles that exist near the metal surface are excited intensely and can be measured as SPF.^27^ The goal of using SPR and SPF is to obtain signal amplification and noise reduction, so that higher analytical sensitivity is achieved compared to conventional immunoassays.^8^ The method has been used widely in research laboratories; however, its clinical application has been limited by the sophisticated technology required for its use.^27^

A novel quantitative BA point-of-care test (Fuji Dri-Chem Immuno AU10V v-BA test; Fujifilm) performed on the point-of-care analyzer (POCA) Immuno AU10V (Fujifilm; hereafter, Fuji refers to the test and analyzer combination, unless otherwise noted) has been developed, applying SPR and SPF for assessment of canine BA. All reactions take place in a small cartridge. The measurand is assessed with a competitive immunoassay using an antigen–antibody reaction that is augmented by SPR and SPF.^16^

There are few published studies focusing on validation of BA measurement in veterinary medicine.^4,5,9,22^ The assay designed for evaluation of BA in humans using the 3GA has been validated for use in dogs and cats.^5^ However, to our knowledge, a study has not been published on validation of the 5GA in veterinary medicine. Overall, reports about evaluation of POCAs for measurement of BA are scarce in veterinary medicine, and only a semiquantitative assay has been reported,^32^ to our knowledge. We evaluated the ease of use and performance characteristics of the Fuji for detection of feline and canine total serum BA concentration compared to the 3GA and 5GA evaluated on a bench-top analyzer, with the 5GA serving as the reference method. Our hypothesis was that the measurement of BA using the Fuji is technically simple and compares well especially with the 5GA for BA.

## Materials and methods

### Study design

Our prospective study was performed between September 2017 and January 2019. Evaluation of performance of the Fuji included assessment of linearity, interferences, lower limit of quantification, intra- and inter-assay precision, and a method comparison study. For the method comparison study, we included serum samples from 60 healthy and diseased cats and 64 dogs submitted to the central diagnostic laboratory, Faculty of Veterinary Medicine, Justus-Liebig-University Giessen, Germany. Patient samples were initially evaluated with the 3GA, which was routinely used in the central laboratory and then assigned to 1 of 3 concentration levels as published previously.^36^ For each species, ~20 samples with normal to mildly increased BA (0–30 µmol/L), 20 samples with moderately increased BA (31–80 µmol/L), and 20 samples with markedly increased BA (>80 µmol/L) were enrolled. The remainder of the sample was then divided into 3 aliquots and frozen at −80°C for up to 8 mo until batch analyzed. Samples were usually processed and frozen within 30 min to 3 h after sample collection. The potential impact of freezing and thawing was assessed by comparing results obtained with the 3GA prior to and after storage at −80°C.

The application of reagents and samples and the measurements were done according to the manufacturer’s instructions and performed by a single trained person on the bench-top analyzer and by 2 trained persons on the Fuji. We performed our study with samples from cats and dogs submitted for routine diagnostic work-up to the central laboratory. Pretreatment of cats and dogs with ursodeoxycholic acid resulted in exclusion from the study because the substance itself is a BA,^34^ and cross-reactivity of this substance with the assay is possible. Also, in another study, it was demonstrated that BA composition changes after treatment with ursodeoxycholic acid.^33^

Our study was conducted in accordance with the German Animal Welfare Act (Article 8). Ethical approval to use excess samples from cats and dogs submitted for routine testing was given by the Regierungspräsidium Giessen, Germany (V54-19c2015h02GI18/17kTV10/2017).

### Measurement with the Fuji POCA

A serum volume of ~300 µL was needed for analysis with the Fuji. Analyses were performed as recommended by the manufacturer. Briefly, cartridges were warmed to room temperature (20–23°C) before opening individual packages, which were then handled carefully without touching the surface. Special care was taken to ensure that the tube with the serum sample was free of air bubbles to avoid analytic error. Calibrations and biochemical reactions were run automatically inside the cartridge. After 10–15 min, the result was displayed on the screen. Results outside the dynamic range stated by the manufacturer for dogs and cats of 2–150 µmol/L were reported as <2 µmol/L or >150 µmol/L.

### Performance characteristics evaluation

#### Method validation

Linearity was assessed as recommended previously^3^ with pooled feline and canine serum spiked with a stock solution containing 0.029 mol/L of bovine-derived sodium cholate hydrate (MilliporeSigma). To avoid matrix effects caused by extensive dilution of serum samples with water, the stock solution was diluted serially so that the same volume of stock solution (10 µL) was always added to the sample (890 µL) to obtain different concentrations. Overall, specimens with BA concentrations of 1.25–100% of the original BA concentration were obtained.

All aliquots spiked with serially diluted stock solutions were analyzed in triplicate with the Fuji. For the sake of comparison, the remainder of the sample spiked with serially diluted stock solution was analyzed once with the 5GA run on a bench-top analyzer (ABX Pentra 400; Horiba). The concentration of BA in the pooled serum was subtracted from the measured concentration of BA in the spiked samples. For all dilution steps, % recovery rate was evaluated by comparing expected and measured results.

Precision was assessed at 3 levels of BA concentration (i.e., low, moderate, and high), whereby 2 samples at each concentration level were included, except for inter-assay variation of pooled cat samples, where only 1 sample of moderate and high concentration levels was available for assessment. Intra- and inter-assay variation was calculated from replicate measurements performed with feline and canine pooled serum samples. For assessment of intra-assay coefficient of variation (CV), 10 replicate measurements were run. Inter-assay CV was calculated from a single BA measurement performed once a day on 7 consecutive days.

For determination of the lower limit of quantification (LLOQ), 2 pooled serum samples with BA concentrations close to zero (cats: 6.4 µmol/L and 2.6 µmol/L, dogs: 6.2 µmol/L and 3.8 µmol/L) were measured 20 times in a single run.

For assessment of the possible impact of lipemia, hemolysis, and hyperbilirubinemia, 960-µL aliquots of pooled feline and canine serum, with a mean concentration of ~5 µmol/L of BA, were spiked with 40 µL of the interfering substance resulting in a concentration of 8 g/L of soybean emulsion (Intralipid 20%; Fresenius Kabi), 4 g/L of hemoglobin (hemoglobin from bovine blood, lyophilized powder; MilliporeSigma), and 800 mg/L of bilirubin (≥98%, powder; MilliporeSigma). The spiked samples were compared with pooled serum samples spiked with equal volumes of the diluent used to prepare the stock solutions of the interfering substance (i.e., pure double-distilled water [in the case of Intralipid], 0.9% NaCl [in the case of hemoglobin], and 100 mM NaOH [in the case of bilirubin]. The stock solutions were prepared as described previously.^19^ The stock solution of the lipid emulsion was further diluted such that a final concentration of 4 g/L (dog) and 2 g/L (cat) was obtained, if necessary. Samples were analyzed in triplicate in random order. Acceptance criterion was that % bias between control and test sample as a result of interferences should be less than the total allowable error (TE_a_) for BA (20%).^18^

#### Method comparison

Patient serum samples stored in 3 aliquots at −80°C were thawed at room temperature before analysis. The results obtained with the Fuji were compared with 2 methods of BA measurement run on a bench-top analyzer (ABX Pentra 400), the 3GA (Colorimetric total bile acid assay; Diazyme Laboratories) and 5GA (LT-SYS Gallensäuren; Labor+Technik). The 5GA run on the Pentra analyzer was considered the reference method.

### Statistical analysis

Software programs (MedCalc v.16.2.1, Ostend; Prism 6, GraphPad) were applied for statistical analysis. The impact of the freeze–thaw cycle on BA concentration was assessed with a Wilcoxon test and a Bland–Altman analysis. A Shapiro–Wilk test was performed to verify the assumption of normality.

Linearity for feline and canine samples spiked with serially diluted bovine BA was investigated for evaluation of the correlation between observed BA values plotted against a calculated (expected) BA concentration. The difference between actual and theoretical BA concentration was used to evaluate recovery after dilution:

$$\mathrm{recovery}\;\%\;=\frac{\mathrm{measured}\;\mathrm{concentration}}{\mathrm{expected}\;\mathrm{concentration}}\times \;100\%$$

Quality requirements for recovery after dilution were set at 80–120% as recommended for immunoassay validation.^2^ Correlation between expected and measured results was assessed with linear and Deming regression analysis. The CV% of the triplicate measurements was determined and % bias between actual (measured concentration) and theoretical (expected concentration) BA concentration was calculated:

$$\%\;\mathrm{bias}=\;\frac{\begin{array}{l} \;\;\;\;{\mathrm{mean}}_{\mathrm{measured}\;\mathrm{concentration}}-\\ \mathrm{expected}\;\mathrm{concentration} \end{array}}{\mathrm{expected}\;\mathrm{concentration}}\;\times \;100\%$$

Imprecision was calculated based on mean and SD:

$$\mathrm{CV}\%\;=\frac{\mathrm{SD}}{\mathrm{mean}}\times \;100\%$$

The mean % difference (d) between test and control sample, and thus the observed interference effect (d_obs_), was determined as follows:

$$d_{\mathrm{obs}}\%=\;\frac{{\mathrm{mean}}_{\mathrm{test}}-{\mathrm{mean}}_{\mathrm{control}}}{{\mathrm{mean}}_{\mathrm{control}}}\;\times \;100\%$$

The d_obs_ % between control sample and sample spiked with interfering substances (hemoglobin, lipid, or bilirubin) should not exceed the TE_a_ of 20%,^18^ and a value less than 0.5 × TEa is desirable.^3^

Correlation and bias between the methods were assessed with Spearman rank analysis, Passing–Bablok regression, and Bland–Altman analysis. Correlations were considered excellent for Spearman rho (*r_s_*) = 0.93–0.99, good for *r_s_* = 0.80–0.92, fair for *r_s_* = 0.59–0.79, and poor for *r_s_* < 0.59.^29^ A Shapiro–Wilk test was used to verify the assumption of normality. Because all results had non-normal distribution, a Kruskal–Wallis test was performed to calculate the difference between median BA concentrations obtained for each analyzer and method. Level of significance was set at *p* ≤0.05 for all analyses. Total observed error (TE_obs_) was assessed and compared with quality specification published previously for BA (i.e., TE_a_).^18^ Quality requirements were fulfilled when TE_obs_ < TE_a_ (TE_a_ = 20%). TE_obs_ = % bias + (2 × CV%).

## Results

### Method validation

The Fuji was simple to use after a short training period. On one study day, continuous use of the analyzer for over 8 h resulted in an error as a result of disturbed machine function, probably caused by build-up of tips. However, the litter bin for tips was only three-quarters filled, and an alert to empty the litter bin had not appeared.

The impact of the freeze–thaw cycle on BA concentration was assessed in 85 serum samples (16 feline, 69 canine). Storage at −80°C did not have a significant impact on BA concentration (*p* = 0.097). There was a minimal mean absolute and % bias of 1.4 µmol/L (1.96 × SD, 18.2 to −15.3 µmol/L) and 2.2% (1.96 × SD, 30.9 to −26.5%), respectively.

**Figure 1.**
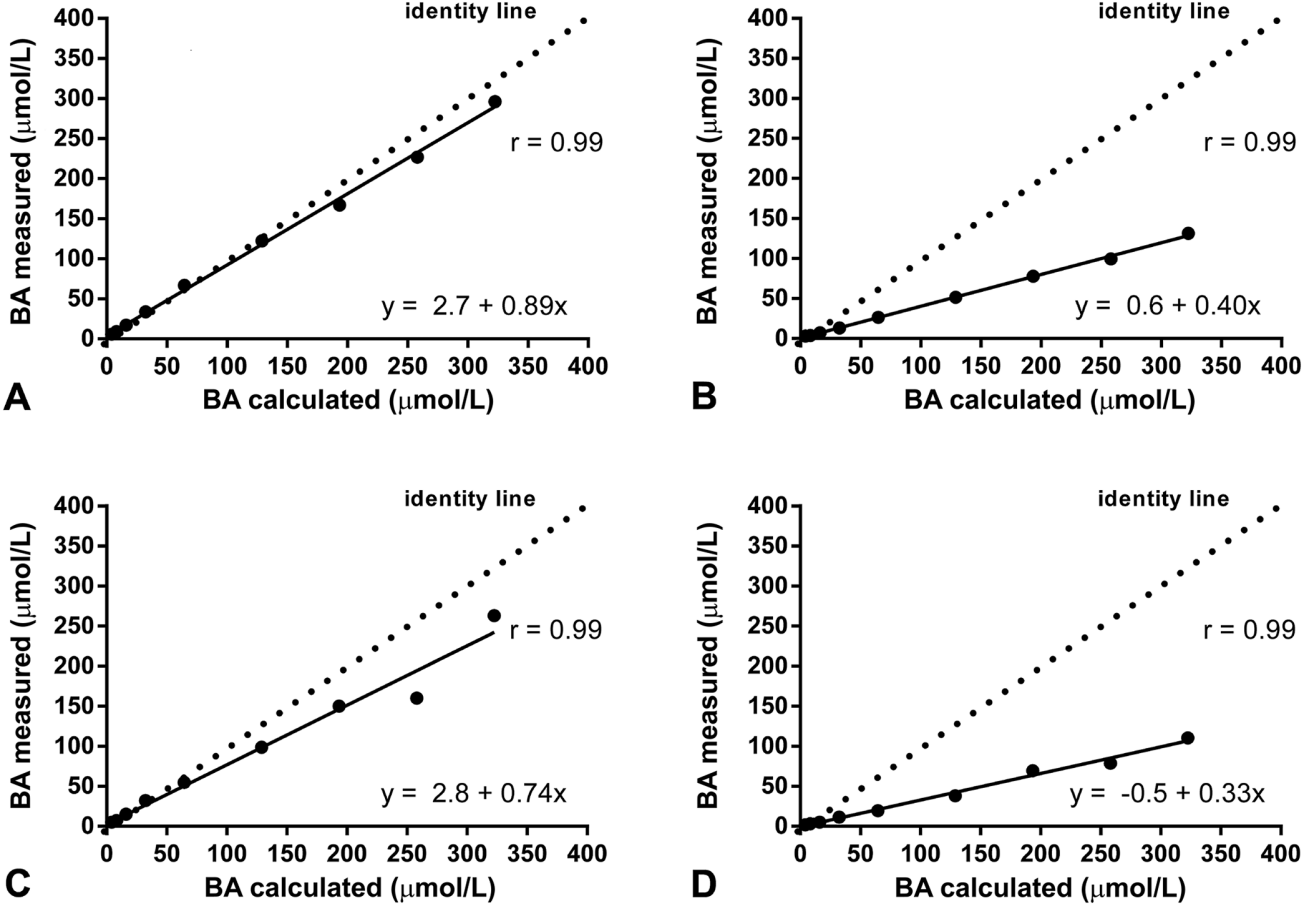
Method validation: linearity of bile acid (BA) measurement of feline (**A**) + (**B**) and canine (**C**) + (**D**) pooled serum (*n* = 9) spiked with 10 µL of serially diluted bovine sodium hydrate cholate with an initial concentration of 323 µmol/L, evaluated with the Fuji Dri-Chem Immuno AU10V point-of-care analyzer (Fujifilm) and a 5th-generation assay (5GA) for bile acids run on the bench-top ABX Pentra 400 analyzer. **A, C.** Linearity under dilution assessed with a 5GA BA assay run on the ABX Pentra 400. **B, D.** Linearity under dilution assessed with the Fuji.

There was excellent correlation between expected and measured results for both cats and dogs, and linearity was demonstrated for a BA concentration up to 130 µmol/L in cats and 110 µmol/L in dogs (Fig. 1, Table 1). However, when feline samples spiked with bovine material were assessed, a marked bias of 26–61% was seen for the Fuji. Similarly, the recovery rate was 39–74% when specimens spiked with bovine BA were assessed (Table 1). Similar results were observed for canine samples (Table 1). Bias was 59–71% and recovery rates were 29–41%. Quality requirements for recovery after dilution could not be fulfilled, and observed bias markedly exceeded the TE_a_ of 20%.

**Table 1.** Method validation: linearity and recovery of bile acids in feline and canine pooled sera with the Fuji Dri-Chem Immuno AU10V point-of-care analyzer.

Dilution factor	Expected concentration (µmol/L)	Mean measured concentration (µmol/L)	Recovery (%)	Bias (%)	CV (%)	TE_obs_ < TE_a_ of 20%
Feline						
0.0125	4.0	3.0	**74**	**26**	1.07	No
0.025	8.1	3.9	**48**	**52**	2.54	No
0.05	16.1	7.2	**45**	**55**	2.80	No
0.1	32.3	13.1	**41**	**59**	0.80	No
0.2	64.5	26.4	**41**	**59**	1.33	No
0.4	129.0	51.5	**40**	**60**	1.81	No
0.6	193.6	77.8	**40**	**60**	1.32	No
0.8	258.1	99.7	**39**	**61**	0.05	No
1	322.6	131.4	**41**	**59**	0.30	No
Canine						
0.0125	4.0	1.7	**41**	**59**	1.31	No
0.025	8.1	3.0	**38**	**62**	0.44	No
0.05	16.1	5.3	**33**	**67**	0.36	No
0.1	32.3	11.5	**36**	**64**	0.42	No
0.2	64.5	19.4	**30**	**70**	1.31	No
0.4	129.0	38.0	**29**	**71**	1.36	No
0.6	193.6	69.5	**36**	**64**	0.70	No
0.8	258.1	78.7	**31**	**70**	3.25	No
1	322.6	110.5	**34**	**66**	0.86	No

CV = coefficient of variation; TE_a_ = total allowable error; TE_obs_ = total observed error. Recovery rates <80% and TE_obs_ > TE_a_ of 20%, are marked in boldface.

Intra-assay CVs calculated from 10 replicate measurements for feline and canine BA at different concentrations were <4% for cats and <5% for dogs (Table 2). In both species, the American Society for Veterinary Clinical Pathology (ASVCP) quality requirements were fulfilled for all concentrations.^18^

**Table 2.** Method validation: intra-assay precision of bile acid measurement in feline and canine pooled sera (*n* = 6) with the Fuji Dri-Chem Immuno AU10V point-of-care analyzer versus 3rd- (3GA) and 5th-generation assays (5GA) for bile acids run on a bench-top analyzer (ABX Pentra 400).

BA conc. range (µmol/L)	Sample ID	Analyzer								
		Fuji (*n* = 10 replicates)			3GA (*n* = 10 replicates)			5GA (*n* = 10 replicates)		
		Mean (µmol/L)	SD (µmol/L)	CV (%)	Mean (µmol/L)	SD (µmol/L)	CV (%)	Mean (µmol/L)	SD (µmol/L)	CV (%)
Feline										
0–30	1	11.7	0.36	3.11	25.4	0.29	1.16	15.0	0.17	1.14
	2	8.2	0.14	1.77	17.8	0.38	2.15	10.0	0.16	1.60
31–80	3	65.4	0.84	1.28	85.4	0.45	0.53	75.2	0.69	0.92
	4	62.8	1.94	3.08	70.7	0.42	0.59	65.6	0.65	0.99
>80	5	108.1	4.20	3.88	127.5	0.81	0.64	118.0	0.91	0.77
	6	94.3	3.09	3,27	110.3	0.66	0.60	101.3	0.83	0.82
Canine										
0–30	1	7.1	0.21	2.96	14.3	0.65	4.53	9.3	0.12	1.24
	2	6.1	0.12	1.91	11.2	0.35	3.10	7.3	0.32	4.37
31–80	3	62.3	0.81	1.31	67.5	0.57	0.84	63.6	0.59	0.92
	4	47.6	0.92	1.94	52.9	0.66	1.25	49.8	0.22	0.44
>80	5	123.3	2.23	1.81	129.8	0.57	0.44	112.1	0.77	0.68
	6	93.5	0.95	1.01	111.4	0.85	0.76	97.8	0.99	1.02

BA = bile acids; conc. = concentration; CV = coefficient of variation.

Intra-assay CVs obtained with the Fuji tended to be lower than those obtained with the Pentra analyzer for both 3GA and 5GA for the low concentration range of canine samples. For both species, CVs in the higher concentration ranges were similar or slightly higher than those obtained with the Pentra analyzer. Inter-assay CVs were <4% for cats and dogs at the 3 different concentrations (Table 3). In both species, quality requirements were fulfilled for all concentrations. Low concentrations of BA (cats ≈ 3 µmol/L, dogs ≈ 4 µmol/L) showed excellent precision, which supports the LLOQ of 2 µmol/L reported by the manufacturer (Table 3).

**Table 3.** Method validation: inter-assay precision of bile acid measurement in 4 feline and 6 canine pooled sera, and intra-assay precision near the lower limit of quantification of bile acids in 2 feline and 2 canine pooled sera with the Fuji Dri-Chem Immuno AU10V point-of-care analyzer.

BA conc. range (µmol/L)	**Cat**	Inter-assay CV (*n* = 7 replicates)			**Dog**	Inter-assay CV (*n* = 7 replicates)		
		Mean (µmol/L)	SD (µmol/L)	CV (%)		Mean (µmol/L)	SD (µmol/L)	CV (%)
0–30	1	2.9	0.10	3.31	1	8.0	0.29	3.65
	2	8.0	0.27	3.39	2	15.0	0.42	2.78
31–80	3	43.0	0.45	1.04	3	48.1	0.77	1.61
					4	53.7	1.28	2.39
>80	4	89.0	1.00	1.13	5	99.3	2.73	2.75
					6	124.5	3.52	2.83
		Intra-assay CV (*n* = 20 replicates)				Intra-assay CV (*n* = 20 replicates)		
	**Cat**	Mean (µmol/L)	SD (µmol/L)	CV (%)	**Dog**	Mean (µmol/L)	SD (µmol/L)	CV (%)
2–4	1	2.63	0.09	3.51	1	3.77	0.14	3.83
≈6	2	6.35	0.15	2.42	2	6.15	0.14	2.21

BA = bile acids; conc. = concentration; CV = coefficient of variation.

Results of the interference experiment (Table 4) showed no effect for hemoglobin at concentrations up to 4 g/L. In contrast, measurement of BA in canine samples spiked with 8 g/L of soybean oil was not possible, and an error was reported by the analyzer. Falsely high BA concentrations were detected when canine samples were spiked with 4 g/L of soybean oil. In feline samples containing the same concentration of soybean oil, measurement of BA was not possible; specimens with 2 g/L of soybean oil had to be prepared, in which BA could be measured with a bias of ~17%, still fulfilling quality criteria. In feline and canine specimens spiked with 800 mg/L of bilirubin, falsely high BA concentrations were detected.

**Table 4.** Method validation: interference testing of bile acid measurement in feline and canine pooled sera spiked with hemoglobin, soybean emulsion, or bilirubin with the Fuji Dri-Chem Immuno AU10V point-of-care analyzer.

Interferent concentration	Mean BA_control_ (µmol/L ± SD)	Mean BA_test_ (µmol/L ± SD)	Bias (µmol/L)	% bias	% bias < TE_a_ of 20%
Cat					
Hemoglobin 4 g/L	5.7 ± 0.12	5.8 ± 0.08	0.07	1.2	Yes
Soybean emulsion 2 g/L	4.9 ± 0.12	5.8 ± 0.12	0.83	16.9	Yes
Bilirubin 800 mg/L	5.7 ± 0.12	7.4 ± 0.46	1.70	29.7	No
Dog					
Hemoglobin 4 g/L	5.7 ± 0.29	5.8 ± 0.16	0.07	1.2	Yes
Soybean emulsion 4 g/L	3.7 ± 0.05	4.6 ± 0.21	0.97	26.4	No
Bilirubin 800 mg/L	5.7 ± 0.00	7.2 ± 0.12	1.47	25.7	No

BA = bile acids; conc. = concentration; TE_a_ = total allowable error. Bias for the interfering substance was considered acceptable if % bias < total allowable error (TE_a_).

### Method comparison

**Figure 2.**
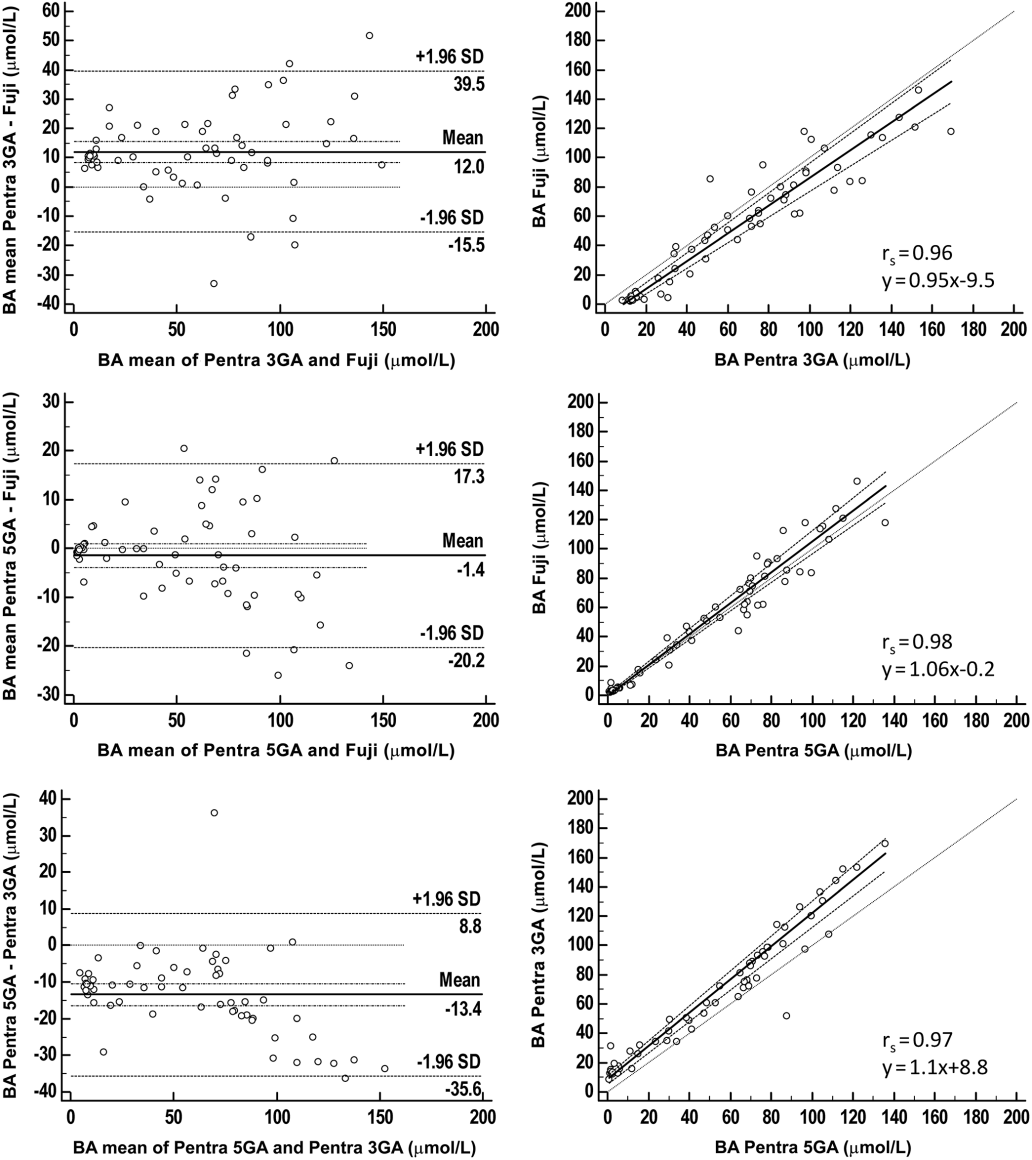
Method comparison of the Fuji Dri-Chem Immuno AU10V point-of-care analyzer (Fujifilm) versus 3rd- (3GA) and 5th-generation assays (5GA) for bile acids (BAs) run on the bench-top ABX Pentra 400 analyzer for 60 feline serum samples. **Left column.** Bland–Altman difference plot demonstrating mean absolute bias (bold line) with 95% confidence interval (CI; small dotted line) and ±1.96 SD limits of agreement (bold dotted lines). **Right column.** Passing–Bablok regression line (solid line) with 90% CI (dotted lines) of BA results assessed by 3 methods. The identity line is the small dotted line.

**Figure 3.**
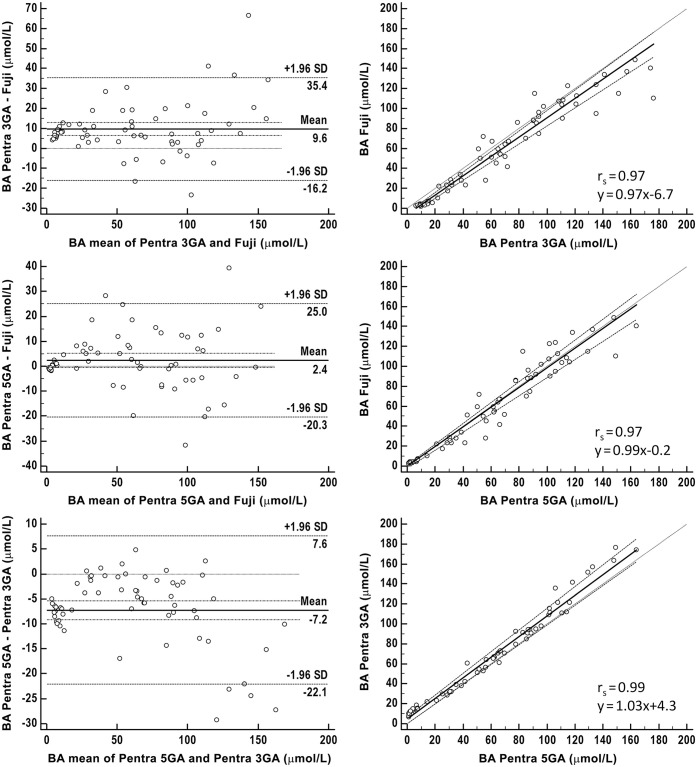
Method comparison of the Fuji Dri-Chem Immuno AU10V point-of-care analyzer (Fujifilm) versus 3rd- (3GA) and 5th-generation assays (5GA) for bile acids (BAs) run on the bench-top ABX Pentra 400 analyzer for 64 canine serum samples. **Left column.** Bland–Altman difference plot demonstrating mean absolute bias (bold line) with 95% confidence interval (CI; small dotted lines) and ±1.96 SD limits of agreement (bold dotted lines). **Right column.** Passing–Bablok regression line (solid line) with 90% CI (dotted lines) of BA results assessed by 3 methods. The identity line is the small dotted line.

**Figure 4.**
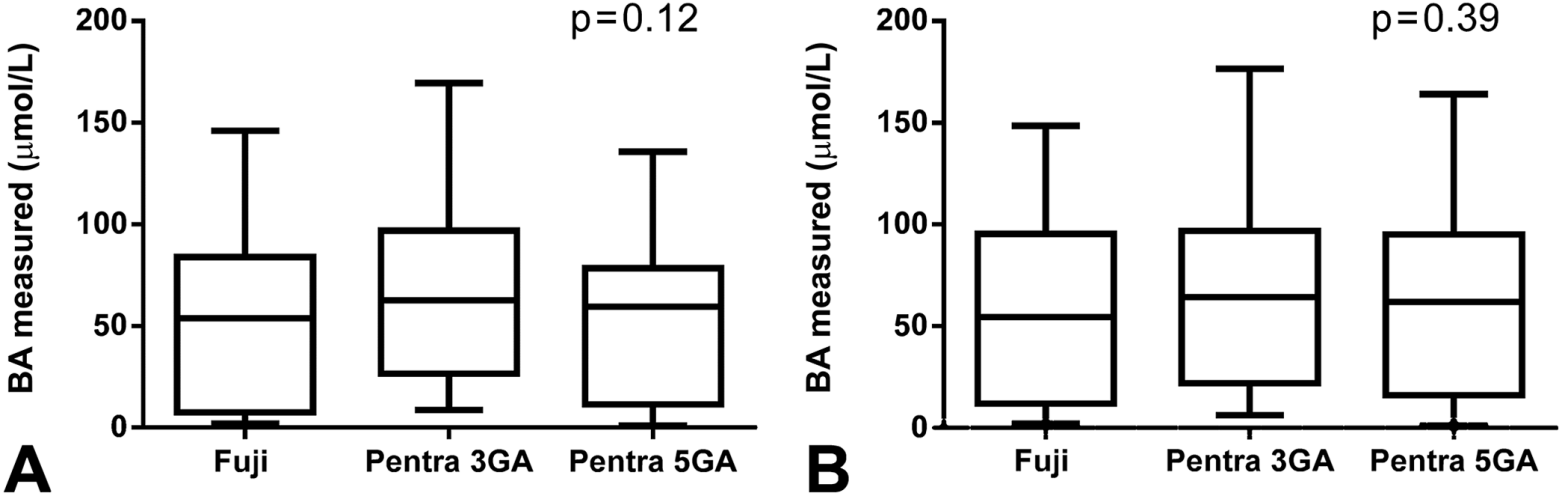
Box-and-whisker diagram demonstrating median and range of the bile acid measurements obtained by 3 methods: Fuji Dri-Chem Immuno AU10V point-of-care analyzer (Fujifilm), and 3rd- (3GA) and 5th-generation assays (5GA) for bile acids run on the bench-top ABX Pentra 400 analyzer, in 60 cats (**A)** and 64 dogs (**B**). The horizontal line in the boxes is the median, the whiskers indicate the range, and the box is the 25th–75th percentile.

We included 60 feline and 64 canine samples. For both species, there was excellent correlation between the methods of 0.96–0.98 in cats (Fig. 2) and 0.97–0.99 in dogs (Fig. 3). Although an absolute mean bias close to zero was present when the Fuji was compared to the 5GA (run on the Pentra analyzer), mean absolute bias was markedly higher when the immunoassay run on the Fuji and the 5GA were compared to the 3GA. Mean absolute bias was 12–14 µmol/L for cats and 7–10 µmol/L for dogs. Overall, mean BA concentrations detected with the 3GA were higher than those assessed with the 5GA and the Fuji. Nevertheless, statistical analysis did not reveal a significant difference between mean BA concentrations obtained with all methods (Fig. 4).

Quality requirements were fulfilled for feline and canine specimens when the Fuji and the 5GA were compared. TE_obs_ was 9–18% in cats and 3–7% in dogs (Table 5), always remaining <20%. These same quality requirements were not fulfilled when the 3GA was compared to the Fuji and the 5GA; TE_obs_ was >TE_a_, irrespective of the analyzer (Table 5).

**Table 5.** Total observed error for bile acids derived from intra-assay precision of 6 pooled serum samples with the Fuji Dri-Chem Immuno AU10V point-of-care analyzer.

BA conc. range (µmol/L)	Sample ID	Analyzer								
		Fuji vs. 3GA (*n* = 10 replicates) Mean bias: 44.1%			Fuji vs. 5GA (*n* = 10 replicates) Mean bias: 6.7%			3GA vs. 5GA (*n* = 10 replicates) Mean bias: 47.7%		
		Mean (µmol/L)	CV (%)	TE_obs_ (%)	Mean (µmol/L)	CV (%)	TE_obs_ (%)	Mean (µmol/L)	CV (%)	TE_obs_ (%)
Cat										
0–30	1	11.7	3.11	**50.3**	11.7	3.11	12.9	25.4	1.16	**50.0**
	2	8.2	1.77	**47.6**	8.2	1.77	10.2	17.8	2.15	**52.0**
31–80	3	65.4	1.28	**46.7**	65.4	1.28	9.3	85.4	0.53	**48.8**
	4	62.8	3.08	**50.3**	62.8	3.08	12.9	70.7	0.59	**48.9**
>80	5	108.1	3.88	**51.9**	108.1	3.88	14.5	127.5	0.64	**49.0**
	6	94.3	3.27	**69.6**	94.3	3.27	18.45	110.3	0.60	**48.9**
		Fuji vs. 3GA (*n* = 10 replicates) Mean bias: 35.3%			Fuji vs. 5GA (*n* = 10 replicates) Mean bias: 1.3%			3GA vs. 5GA (*n* = 10 replicates) Mean bias: 32.3%		
		Mean (µmol/L)	CV (%)	TE_obs_ (%)	Mean (µmol/L)	CV (%)	TE_obs_ (%)	Mean (µmol/L)	CV (%)	TE_obs_ (%)
Dog										
0–30	1	7.1	2.96	**41.2**	7.1	2.96	7.2	14.3	4.5	**41.4**
	2	6.1	1.91	**39.1**	6.1	1.91	5.1	11.2	3.1	**38.5**
31–80	3	62.3	1.31	**37.9**	62.3	1.31	3.9	67.5	0.84	**34.0**
	4	47.6	1.94	**39.2**	47.6	1.94	5.2	52.9	1.25	**34.8**
>80	5	123.3	1.81	**38.9**	123.3	1.81	6.2	129.8	0.44	**33.2**
	6	93.5	1.01	**37.3**	93.5	1.01	3.3	111.4	0.76	**33.8**

BA = bile acids; conc. = concentration; CV = coefficient of variation; TE_a_ = total allowable error; TE_obs_ = total observed error. TE_obs_ > TE_a_ (20%; i.e., results not fulfilling quality requirements are shown in boldface). Mean bias = bias results of the method comparison study in 60 feline and 64 canine samples.

## Discussion

Overall, the Fuji v-BA assay was simple to use and fulfilled all quality requirements, based on ASVCP guidelines on TE_a_,^18^ when compared to the 5GA run on the Pentra analyzer. Marked bias was present comparing the 3GA for BAs to the 5GA and the Fuji, exceeding quality requirements for BA in veterinary medicine.^18^ The bias was also seen when the 3GA and the 5GA were both run on the Pentra analyzer, indicating that the method rather than the analyzer was the source.

When a sample spiked with bovine material was assessed, a marked bias of 26–71% in linearity was seen for the Fuji. This bias might be attributed to the different composition of the total BA pool in different mammals,^20,34^ and indicates the species-specificity of the antibody used for the Fuji v-BA assay. It is also not uncommon that enzymatic assays have variable recovery of some types of BA.^13,39^ In one study comparing 3 different enzymatic assays for measurement of total BA to liquid chromatography–mass spectrometry, all commercial enzymatic assays substantially underestimated the total BA with a bias of −12 to −44%.^10^ The results of that study and our study underline the importance of species-, analyzer-, and assay-specific reference intervals.

A minor limitation of the Fuji is its limited dynamic range of 2–150 µmol/L BA. Results outside of that range are reported as <2 µmol/L or >150 µmol/L. Dilution of the samples to quantify results >150 µmol/L is not possible because the viscosity of the sample has an important influence on the performance of the analyzer and could lead to an erroneous measurement.^16^ However, this limited dynamic range has only minor clinical relevance given that a broad concentration range is reportable. Quantitative results at a higher concentration might be relevant for follow-up examinations to evaluate trends. Samples with BA concentrations exceeding the dynamic range of the Fuji should be assessed with the 5GA run on a Pentra analyzer if follow-up examinations are relevant for the patient. The Fuji is superior to the only alternatively available POC test for BA (Snap bile acids test; Idexx), which can only quantify BA concentrations of 5–30 µmol/L.^32^

The evaluation of the LLOQ was also impaired by BA concentrations <2 µmol/L. However, evaluation of serum samples with BA concentrations close to this lower detection limit showed low intra-assay CVs <4% and fulfilled quality requirements.

For all methods and both analyzers, intra-assay CVs fulfilled quality requirements. Interestingly, in dogs, intra-assay CV of the Fuji tended to be lower in the low concentration range, whereas the contrary was observed for the tests run on the Pentra analyzer. A higher CV is often observed at lower concentrations than at higher ranges. The relatively high intra-assay precision of the Fuji at low concentrations might be attributed to the high analytical sensitivity of the novel method using SPR and SPF.^26^ Additionally, all quality requirements were met when inter-assay CV was evaluated with the Fuji. A limitation of our replication study is that only 10 measurements were performed to calculate intra- and inter-assay CV given limitations on serum volume, whereas 20 replications are recommended to evaluate precision.^14^ However, a minimum of 5 replications is considered acceptable.^15,25,30^

Hyperbilirubinemia at a concentration of 800 mg/L and lipemia at concentrations >2 g/L caused falsely high results with the Fuji. We did not evaluate interferences on the 5GA; however, a previous study of human samples found no interferences for the enzyme cycling methods at concentrations of 680 µmol/L bilirubin (~398 mg/L), 5.0 g/L hemoglobin, or 11.3 mmol/L triglyceride (~9.9 g/L).^39^ In contrast, one manufacturer provides information of a minor interfering effect below 10% bias of triglycerides in people (i.e., at a concentration of 7.5 g/L, hemoglobin of 5 g/L, or bilirubin of 500 mg/L).^11^ A limitation in our study is that interferences were evaluated at mean BA concentrations of 3.7–5.7 µmol/L. Evaluation of interferences at a clinically relevant concentration range is preferable (i.e., the upper reference range, where falsely high results might lead to a different clinical decision or a different treatment plan). In our study, a relatively small number of patients had high BA concentrations, thus an insufficient volume of pooled plasma was available to perform interference studies at different concentrations. At higher concentrations, the impact of bilirubin and lipid might have resulted in lower % bias, possibly still fulfilling quality criteria, and warrants further study. The manufacturer tested possible interferences and claims no significant effect for bilirubin at 340 µmol/L, which is at a lower concentration level than tested in our study (800 mg/L, ~1,386 µmol/L).^16^ Moreover, the manufacturer declares no effect for hemoglobin at 4,000 mg/L, which we confirmed in our study. In contrast to our study, there was no impact of lipemia (named “chyle”) on BA measurement reported by the manufacturer at a concentration level of 2,000 FTUs (formazin turbidity units). However, results are not directly comparable with our findings given a different method and unit. Users should be aware of possible interferences in clinically relevant hyperbilirubinemia and hypertriglyceridemia and consider that measurement of severely lipemic samples may not be possible. The interfering effect of bilirubin on BA measurement is considered of minor clinical relevance, given that the assessment of BA is generally not recommended in patients with hyperbilirubinemia.^24^

A potential limitation of our method validation study is that serum samples were frozen at −80°C for up to 8 mo until batch analyzed. Evaporation or other alterations of the BA concentration might occur during freeze–thaw cycles. In fact, a study that investigated the effects of storage time and freezing temperature on canine serum BA concentration reported a mean difference from baseline >10%, with possible clinical relevance; however, the mean BA concentration was quite low (1.7 µmol/L), and higher CVs at low concentrations make interpretation of bias difficult.^35^ The impact of freezing on our BA results was negligible, as reflected by a small bias of 2.2%. Our results are in accordance with studies in human medicine reporting stability of BA during freezing over several months.^21,37^ Given that all aliquots analyzed with the different methods underwent the same freeze–thaw cycle, possible freezing-induced alterations would have affected all samples in the same manner and are thus considered of minor relevance in our study.

For internal quality control, a pooled serum sample is recommended by the manufacturer. Unfortunately, control material is not provided by the manufacturer, and commercially available control material for BA assays is of limited use for the Fuji. This is attributed to the control material that might be artificial and exhibit different viscosity characteristics that are not suitable for the analysis with the Fuji, or material might contain BA from other species such as bovine BA, with low recovery rates. The use of a pooled serum sample that additionally must be adjusted in accordance with clinically significant levels seems rather complicated and impractical, especially for in-house usage of the Fuji, which makes detection of deviation from stable analyzer performance difficult for the user.
